# Video-Mosaicked Handheld Dual-Axis Confocal Microscopy of Gliomas: An *ex vivo* Feasibility Study in Humans

**DOI:** 10.3389/fonc.2020.01674

**Published:** 2020-08-27

**Authors:** Yoko Fujita, Linpeng Wei, Patrick J. Cimino, Jonathan T. C. Liu, Nader Sanai

**Affiliations:** ^1^Ivy Brain Tumor Center, Barrow Neurological Institute, St. Joseph’s Hospital and Medical Center, Phoenix, AZ, United States; ^2^Department of Mechanical Engineering, University of Washington, Seattle, WA, United States; ^3^Department of Pathology, University of Washington, Seattle, WA, United States; ^4^Department of Bioengineering, University of Washington, Seattle, WA, United States; ^5^Department of Neurosurgery, Barrow Neurological Institute, St. Joseph’s Hospital and Medical Center, Phoenix, AZ, United States

**Keywords:** confocal microscopy, fluorescence, glioma, surgery, video mosaic

## Abstract

**Background:**

Intraoperative confocal microscopy can enable high-resolution cross-sectional imaging of intact tissues as a non-invasive real-time alternative to gold-standard histology. However, all current means of intraoperative confocal microscopy are hindered by a limited field of view (FOV), presenting a challenge for evaluating gliomas, which are highly heterogeneous.

**Objective:**

This study explored the use of image mosaicking with handheld dual-axis confocal (DAC) microscopy of fresh human glioma specimens.

**Methods:**

In this preliminary technical feasibility study, fresh human glioma specimens from 6 patients were labeled with a fast-acting topical stain (acridine orange) and imaged using a newly developed DAC microscope prototype.

**Results:**

In comparison to individual image frames with small fields of view, mosaicked images from a DAC microscope correlate better with gold-standard H&E-stained histology images, including the ability to visualize gradual transitions from areas of dense cellularity to sparse cellularity within the tumor.

**Conclusion:**

LS-DAC microscopy provides high-resolution, high-contrast images of glioma tissues that agree with corresponding H&E histology. Compared with individual image frames, mosaicked images provide more accurate representations of the overall cytoarchitecture of heterogeneous glioma tissues. Further investigation is needed to evaluate the ability of high-resolution mosaicked microscopy to improve the extent of glioma resection and patient outcomes.

## Introduction

Extent of resection is one of the strongest predictors of overall survival, progression-free survival, and quality of life in both low-grade (1–5) and high-grade (6, 7) gliomas (8). Achieving extensive microsurgical resection while trying to preserve neurological function, however, remains a challenge due to the infiltrative nature of these tumors and the difficulty in distinguishing tumor regions from adjacent normal brain tissues based on gross tissue characteristics.

In recent decades, several intraoperative imaging techniques have been developed to improve glioma resection. For example, neuronavigation based on preoperative magnetic resonance imaging (MRI) is now the standard of care, but this technique is often inaccurate due to brain shift caused by cerebrospinal fluid loss, cerebral edema, and resection-induced deformations. While intraoperative MRI circumvents brain-shift artifacts, it is not commonly used in routine clinical practice due to its high costs and prolonged surgical times. Intraoperative ultrasonography (9) can be more time-effective and cost-effective than intraoperative MRI, but it offers limited resolution and contrast, which limits sensitivity and specificity. Fluorescence image-guided surgery (FIGS) utilizing conventional wide-field surgical microscopy is another clinically accepted technique for neurosurgical guidance and has been shown to be an effective method to improve the extent of resection in high-grade gliomas (10).

In neurosurgery, fluorescent dyes such as 5-ALA, sodium fluorescein, indocyanine green (ICG) have been used to guide removal of brain tumors in human (10–14). Details for these fluorescent contrast agents, as well as others, have been published by others and are beyond scope of this paper. However, it is worth noting that only 5-ALA has been approved for guiding high-grade glioma resections by the US Food and Drug Administration (FDA). 5-ALA is a non-fluorescent precursor which is metabolized into an endogenous fluorophore, protoporphyrin IX (PpIX), which accumulates selectively in tumor cells versus normal cells (15–18).

However, a fundamental limitation to FIGS, which typically utilizes low-resolution (i.e., low-power) fluorescence microscopy, is the limited sensitivity to detect the weak fluorescence generated by low-grade tumor cells, as well as the sparse signal from infiltrative disseminated tumor cells (e.g., at the margins of all diffuse gliomas) (16, 19, 20). In current clinical practice, intraoperative frozen-section analysis is often used for preliminary tumor diagnosis and to assess the tumor margin for surgical decision-making. However, frozen-section analysis is time-consuming and prone to processing and imaging artifacts, such that biopsies can be inconclusive and additional sampling is required (21).

Intraoperative confocal microscopy is a high-resolution optical imaging technique that uses a spatial filter (e.g., a pinhole or detection slit) to reject out-of-focus and multiply scattered light in tissue. It can provide cross-sectional images of intact tissue (i.e., optical sectioning) as a non-invasive real-time alternative to slide-mounted histology for guiding glioma resections. Several commercial intraoperative confocal microscope devices have been implemented in previous studies (22, 23). In 2011, Sanai et al. (24) reported the first *in vivo* human study demonstrating the feasibility of this technology for guiding brain tumor resections. Many other preclinical and clinical studies have also been performed to demonstrate the clinical potential of intraoperative confocal microscopy, including for visualizing gliomas in mouse models (25, 26), identifying histological features in different types of brain tumors (21, 23), and for diagnosing human brain tumors (27). While early studies have yielded promising results, all miniature confocal microscope systems have a limited field of view (FOV), requiring the user to mentally integrate many images to assess overall tissue architecture and accurately identify pathological landmarks. This is especially challenging in gliomas, which are highly heterogeneous.

We have recently developed a handheld confocal microscope, based on a line-scanned dual-axis confocal (LS-DAC) architecture, which can provide video-rate (>16 Hz) imaging with subnuclear resolution (28–30). In particular, recent technical advances now enable image-mosaicking capabilities (i.e., generating an extended FOV over time using image-processing algorithms) (29). With image mosaicking, a sequence of adjacent images is acquired rapidly as the handheld microscope is slowly translated across a tissue surface, such that the overlapping image frames are stitched together to create a larger panoramic image. In practice, a high-frame-rate microscopy device is needed, both to minimize motion artifacts within each image frame and to ensure sufficient overlap between each adjacent image frame (for feature-based image mosaicking) as the device is slowly translated by hand across the tissue surface. In an early preclinical study using acridine-orange-stained fresh mouse tissue, we showed that our device (where each image frame has an FOV of ∼350 × 350 μm) was able to visualize both the microscopic structures (e.g., subnuclear features) and macroscopic features (e.g., glands and tubules) over a total mosaicked imaging field of >2 mm (29).

Here, we report our first experience using a handheld LS-DAC microscope with image mosaicking to visualize fresh human glioma tissues resected from human patients.

## Materials and Methods

### Patient Selection

Patients with gliomas undergoing craniotomy for tumor resection were considered as candidates for the study. Patients younger than 18 years of age were excluded. Preoperative informed consent was obtained for all patients.

### Tissue Acquisition and Processing

During surgery, biopsies were taken for routine histopathological analysis, and per the IRB protocol, part of the biopsy was collected for our study. Tumor specimens were immediately wrapped by 1× phosphate-buffered saline (PBS)-soaked gauze, stored on ice in a sealed container, and then shipped to the University of Washington with overnight freight services. Upon receipt, the tumor specimen was topically stained with 1mM acridine orange (A6014, Sigma-Aldrich Inc., St. Louis, MO, United States) for 1 min, and was then washed 3 times in PBS to remove the unbound fluorescent agent. The tissue surface was imaged with the handheld LS-DAC microscope at 16 frames per second (fps). The tissue was subsequently fixed in 10% formalin and submitted for hematoxylin and eosin (H&E) histology. Paraffin-embedded tissues were physically sectioned (4-μm thickness) in the en face direction as close and parallel to the imaged tissue surface as possible. Slide-mounted sections were stained with H&E and imaged by a board-certified neuropathologist (PC) to identify hallmarks of glioma.

### Line-Scanned Dual-Axis Confocal Microscope

The imaging system used in this study is based on the device described in a recent publication (29). In brief, the handheld imaging probe has an outer diameter of 14 mm and a small contact window of 5 mm. The device is capable of providing an FOV of approximately 350 μm by 350 μm with a lateral resolution of approximately 1 μm and an axial resolution of <2 μm in brain tissue up to ∼150-μm deep. In this study, we utilize a 488-nm blue diode laser (Omicron LuxX 488, Rodgau-Dudenhofen, Germany) with a maximum power of 1 mW (at the tissue) to excite fluorescence (acridine orange) within the tissue specimen. All images in this work were acquired at 16 fps and displayed on a computer monitor in real time. It should be noted that the frame rate can be further increased if necessary, at the cost of reduced signal-to-noise ratio (SNR). However, 16 Hz was an optimal frame rate to effectively reduce motion artifacts during handheld use while retaining a sufficient SNR in this study. In addition, we used a lens cap optimized to provide a constant imaging depth at approximately 50 μm beneath the tissue surface. A set of lens caps are available to enable the device to image at different depths ranging from 0 to 150 μm, and these lens caps can be interchanged during imaging sessions. Importantly, enabled by the high frame rate and lens cap design (newly optimized), the device provides smooth and continuous images and is, therefore, capable of video mosaicking – stitching overlapping video frames to create an extended FOV over time using image processing algorithms – to sample a tissue region similar in size to a physical biopsy specimen (i.e., a few millimeters in scale). The raw video clips from the LS-DAC microscope are processed into large mosaics using Image Composite Editor (Microsoft Inc., Redmond, WA, United States) using default settings. The probe could be operated as either a handheld unit or mounted onto a mechanical system (such as a translation stage or a Greenberg retractor) for localization of the probe tip.

## Results

### Patient Demographics

Six patients were enrolled in the study (3 men, 3 women: age range, 20–87 years). All patients had glioma-suspected mass lesions requiring neurosurgical intervention. They were treated by one attending neurosurgeon (NS) at the Barrow Neurological Institute from May 2019 to September 2019. Tumor specimens were obtained from enrolled patients. All 6 lesions were diagnosed as gliomas, in which 4 were World Health Organization (WHO) grade IV, 1 was grade III, and 1 was grade I diffuse gliomas (Table 1).

**TABLE 1:** Patient characteristics.

Case no.	Histopathology	Grade	Age/sex	Tumor location
1	Pilocytic astrocytoma	I	20/M	Temporal
2	Glioblastoma	IV	71/M	Frontal
3	Anaplastic astrocytoma	III	38/F	Temporal
4	Glioblastoma	IV	36/F	Temporal
5	Glioblastoma	IV	41/F	Temporal
6	Glioblastoma	IV	87/M	Parietal

*M, male; F, female.*

### LS-DAC Microscopy of Human Gliomas Stained With Acridine Orange

**FIGURE 1:**
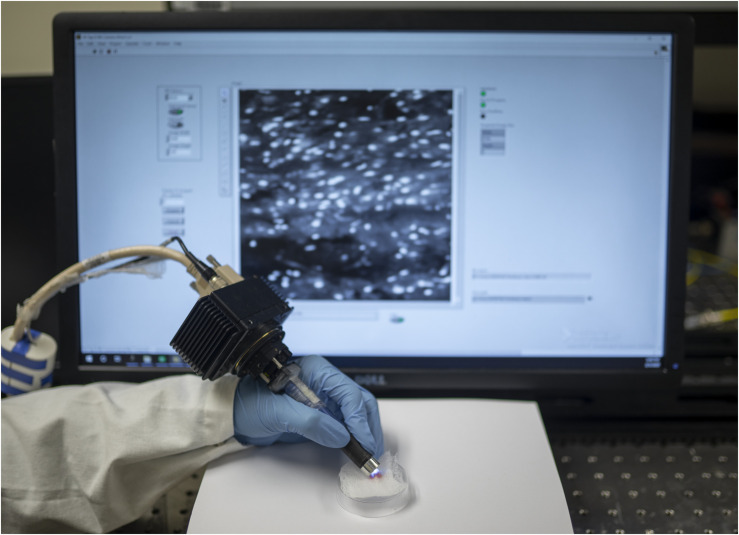
Hand-held dual-axis confocal (DAC) microscope system. As described in a previous publication [L. Wei et al., *Opt. Lett.*, 2019], the DAC microscope allows for high-speed (16 frames/sec) optical-sectioning fluorescence microscopy of intact thick tissues. The spatial resolution of the images is at the subnuclear scale (∼1 μm lateral, ∼2 μm axial), and the field of view of each image frame is ∼350 × 350 μm. Interchangeable lens caps enable fixed imaging depths at up to ∼150 microns beneath the tissue surface.

**FIGURE 2:**
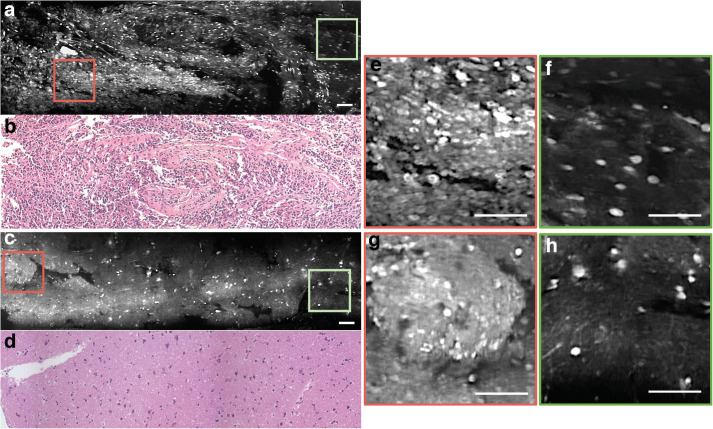
Mosaicked images of human gliomas. **(a)** Mosaicked LS-DAC image of a human glioma with hypercellularity, along with corresponding H&E histology **(b)**. **(c)** Mosaicked LS-DAC image of human glioma with overall normal cellularity and cytoarchitecture, along with corresponding H&E histology **(d)**. While the large-area mosaics provide a non-ambiguous assessment of the two specimens, individual image frames from the glioma specimen **(e,f)** can present similar characteristics to those from the benign specimen **(g,h)**. Scale bars represent 100 μm. LS-DAC, line-scanned dual-axis confocal; H&E, hematoxylin and eosin.

The handheld LS-DAC microscope used in this study is shown in Figure 1. Tissue integrity was maintained during confocal microscopy and histopathological analysis, as demonstrated by the excellent preservation of cellular and subcellular structures. In both normal and tumor regions, it was possible to identify nuclei with higher acridine orange signal compared to that of the cytoplasm and neuropil (Figures 2a–h). There were minimal motion artifacts seen in the images as the LS-DAC microscope was translated by hand along various lateral directions across various tissue surfaces. Due to the heterogeneity of most tumors at the sub-millimeter scale (and even at centimeter scales), individual image frames can be misleading and ambiguous (Figures 2e–h). Mosaicked images, which provide several millimeters of spatial coverage, provide a more accurate and representative view of gliomas that correlate better with gold-standard H&E histology images (Figures 2a,c), including the ability to visualize gradual transitions from areas with dense cellularity to sparse cellularity within the tumor.

### Image Atlas for Mosaicked LS-DAC Microscopy of Gliomas Labeled With Acridine Orange

**FIGURE 3:**
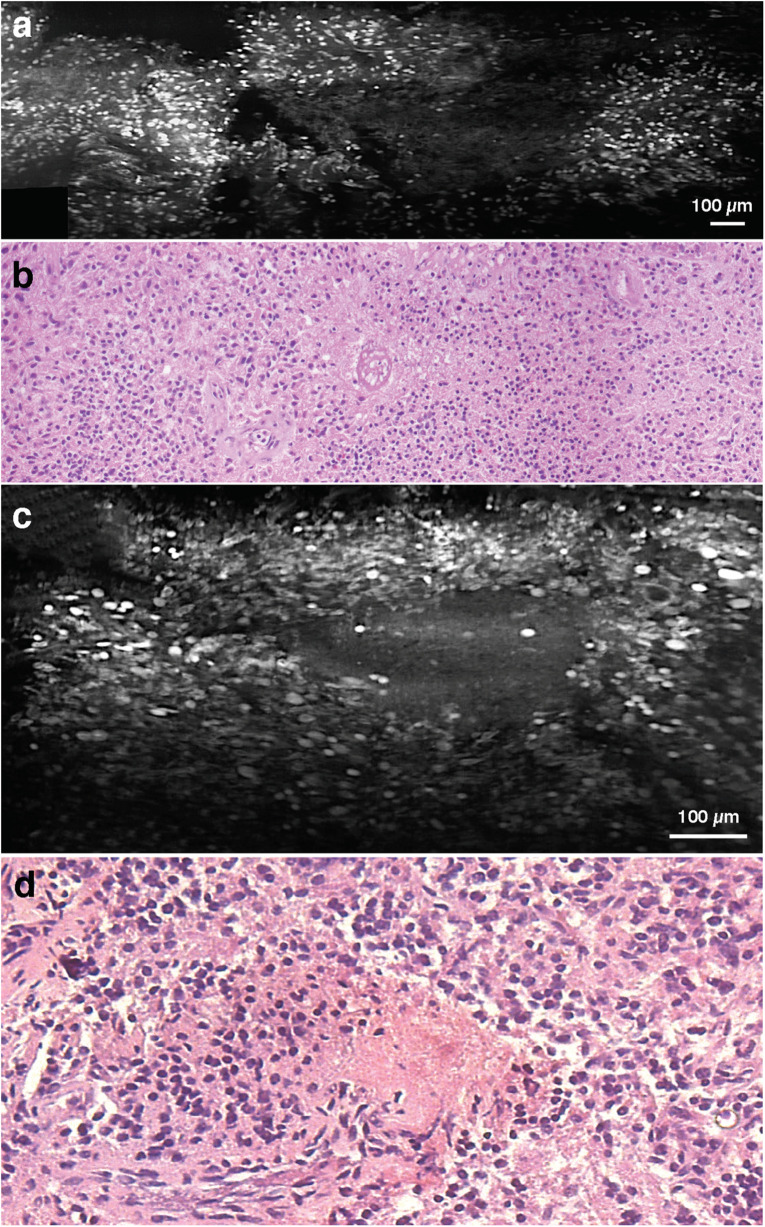
Necrosis. **(a)** LS-DAC image showing necrotic regions in human gliomas (topically stained with acridine orange), along with **(b)** corresponding H&E histology. **(c)** Additional LS-DAC image of necrotic regions in human gliomas stained with acridine orange with **(d)** corresponding H&E histology. LS-DAC, line-scanned dual-axis confocal; H&E, hematoxylin and eosin.

**FIGURE 4:**
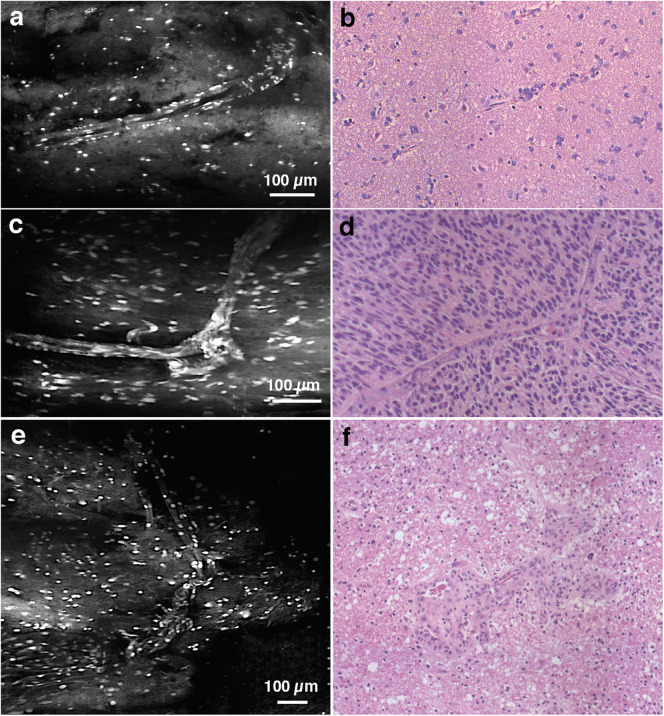
Vasculature. **(a)** LS-DAC image showing the vasculature in human gliomas (topically stained with acridine orange) with **(b)** corresponding H&E histology. **(c–f)** Additional LS-DAC images of human glioma vasculature acridine orange staining with corresponding H&E histology. LS-DAC, line-scanned dual-axis confocal; H&E, hematoxylin and eosin.

**FIGURE 5:**
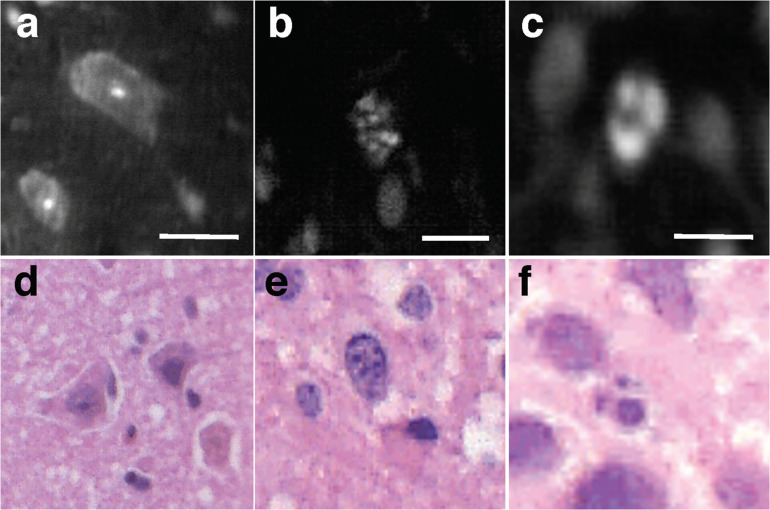
Subnuclear features. Examples of LS-DAC images showing normal neurons **(a)**, and subcellular features with different chromatin patterns **(b,c)** in human gliomas (topically stained with acridine orange), along with corresponding H&E histology **(d–f)**. Scale bars represent 10 μm. LS-DAC, line-scanned dual-axis confocal; H&E, hematoxylin and eosin.

Among WHO grade III and IV diffuse gliomas, LS-DAC images reveal higher cellular density, nuclear atypia, and microvascular proliferation (Figure 2). In grade IV gliomas, histopathological hallmarks such as necrosis surrounded by palisading tumor cells (Figure 3) or microvascular proliferation (Figure 4) can also be identified. Those features were better identified via mosaicked imaging than via individual image frames, and they correlated better with corresponding H&E histology images. Interestingly, the high-resolution and high-contrast imaging capability of the LS-DAC microscope enabled the visualization of neurons and various subnuclear chromatin patterns (Figure 5).

## Discussion

Mounting evidence supports the value of the safe maximal extent of resection in glioma surgery. Intraoperative techniques to guide glioma resections include intraoperative neuro-navigation based on preoperative MRI, intraoperative MRI, intraoperative ultrasonography, and FIGS. However, these low-resolution imaging techniques often lack the sensitivity and/or specificity to reliably detect diffuse glioma cells, especially at the infiltrative surgical margins. In recent decades, intraoperative high-resolution confocal microscopes have been developed for surgical guidance and allow for the sensitive detection of glioma cells beyond the radiographic margins (31). However, as mentioned previously, one of the primary motivations for this study is the realization that the limited FOV of intraoperative high-resolution microscopes (typically <0.5 mm by 0.5 mm) can lead to ambiguous and misleading results, especially since most tumors exhibit significant spatial heterogeneity at sub-millimeter scales. Diffuse gliomas are particularly heterogeneous, in which infiltrating tumor cells will often migrate over centimeter scales (32). Therefore, there is a need for a high-resolution imaging strategy that can provide larger imaging fields in which pathological features can be reliably visualized and spatial gradients can be recognized (e.g., changes in cellular density).

In addition to the small sample size of this technical feasibility study, there are several other limitations. First, this study was performed with fresh *ex vivo* glioma tissues. Future studies will aim to demonstrate the value of this device for guiding glioma resections in humans *in vivo*. The ideal study would incorporate our imaging technologies for guiding surgical decisions and would assess resulting surgical outcomes such as the extent of resection. Another shortcoming of the present study is that acridine orange is not approved for *in vivo* use in patients. As an alternative, we are exploring the development and clinical validation of a handheld optical-sectioning device to image and quantify the expression of 5-ALA-induced PpIX, which preferentially accumulates in proliferative tumor cells as described in the introduction and in a recent perspective article (20). In the study described here, patient specimens were mostly obtained from bulk tumor regions, and may not reflect the pathological characteristics at the infiltrating edge of gliomas. Future studies are needed to assess the ability to detect and quantify fluorescence contrast from the sparse disseminated cell populations at the surgical margins.

## Conclusion

This report describes our first experience with using a handheld LS-DAC microscope, with image mosaicking, to image fresh human glioma tissues resected from human patients. Our results show that LS-DAC microscopy provides high-resolution, high-contrast images of glioma tissues topically stained with acridine orange, with subnuclear resolution, that agree with corresponding H&E histology. The image-mosaicking capability of the LS-DAC microscope provides more accurate representations of the overall cytoarchitecture of heterogeneous glioma tissues that are in better agreement with standard histology images. Further investigation is needed to evaluate its ability to detect and quantify fluorescence contrast at the surgical margins to improve the extent of resection of gliomas.

## Data Availability Statement

All datasets presented in this study are included in the article/supplementary material.

## Ethics Statement

The studies involving human participants were reviewed and approved by the St. Joseph’s Hospital and Medical Center’s Institutional Review Board (IRB No. 17-0378-30-12). The patients/participants provided their written informed consent to participate in this study. Written informed consent was obtained from the individual(s) for the publication of any potentially identifiable images or data included in this article.

## Author Contributions

JL and NS: conception, design, and critically revising the manuscript. YF and LW: acquisition of the data and drafting the manuscript. YF, LW, and PC: analysis and interpretation of the data. All authors contributed to the article and approved the submitted version.

## Conflict of Interest

The authors declare that the research was conducted in the absence of any commercial or financial relationships that could be construed as a potential conflict of interest.
